# 79 Factors Predicting Burn Survivor Group Interest in an Outpatient Burn Clinic

**DOI:** 10.1093/jbcr/irac012.082

**Published:** 2022-03-23

**Authors:** Erin E Ross, Rachel A Colbath, Jeremy Yu, Naikhoba Munabi, Justin Gillenwater, Haig A Yenikomshian

**Affiliations:** Keck School of Medicine, University of Southern California, Los Angeles, California; Keck School of Medicine of USC, Carlsbad, California; University of Southern California, Los Angeles, California; Keck school of medicine of USC, Los Angeles, California; USC/LAC+USC Medical Center, Los Angeles, California; USC/LAC+USC Medical Center, Los Angeles, California

## Abstract

**Introduction:**

Outpatient peer support groups have been demonstrated to help patients reintegrate with the community and improve self-acceptance following burn injury. Despite this, there is little data examining who desires participation. This is especially true for minority and socio-disadvantaged populations. The purpose of this study is to examine factors that influence desire to participate in burn survivor groups and actual participation rates.

**Methods:**

Patients attending outpatient clinic were asked about participation in burn survivor group, interest in joining a group, and administered National Institute of Health Patient-Reported Outcomes Measurement Information System (PROMIS) Managing Emotions (ME) and Managing Social Interactions (MSI) questionnaires. Patient demographics, total body surface area burned (TBSA), initial hospital length of stay (LOS), and surgical intervention were collected from the medical record. While controlling for age and gender, indicators of injury severity and scores on PROMIS questionnaires were each examined for associations with survivor group interest using Firth or standard binary logistic regression.

**Results:**

70 patients completed surveys in English (60%, 42/70) and Spanish (40%, 28/70). Current or past participation in burn survivor group was low (n=3/70, 4.2%), with greater interest in joining burn survivor group (n=20/66, 30.3%). Most patients interested in burn survivor group were Spanish speaking (65%, 13/20). Interest in joining a burn survivor group was associated with all collected measures of injury severity (Table 1). Scores on ME and MSI were not significantly associated with burn survivor group interest (ME p=.636, MSI p=.133).

**Conclusions:**

There is a gap between interest and participation in burn survivor group, particularly among Spanish speaking patients. Patients with increase in TBSA, LOS, and undergoing surgery were more likely to express interest in burn survivor group participation. Interestingly, patients’ self-reported outcomes on emotional distress and interactions with others were not related to interest in burn survivor group.

